# Ethical considerations around volunteer payments in a malaria human infection study in Kenya: an embedded empirical ethics study

**DOI:** 10.1186/s12910-022-00783-y

**Published:** 2022-04-20

**Authors:** Primus Che Chi, Esther Awuor Owino, Irene Jao, Philip Bejon, Melissa Kapulu, Vicki Marsh, Dorcas Kamuya

**Affiliations:** 1 Centre for Geographic Medicine Research (Coast), Kenya Medical Research Institute-Wellcome Trust Research Programme, Kilifi, Kenya; 2 Nuffield Department of Medicine, Centre for Tropical Medicine and Global Health, University Oxford, Oxford, UK

## Abstract

Human Infection Studies (HIS) have emerged as an important research approach with the potential to fast track the global development of vaccines and treatments for infectious diseases, including in low resource settings. Given the high level of burdens involved in many HIS, particularly prolonged residency and biological sampling requirements, it can be challenging to identify levels of study payments that provide adequate compensation but avoid ‘undue’ levels of inducement to participate. Through this embedded ethics study, involving 97 healthy volunteers and other research stakeholders in a malaria HIS programme in Kenya, and using in-depth interviews, focus group discussions and observations during and after a malaria HIS, we give a grounded account of ethical issues emerging in relation to study payments in this setting. While careful community, national, international scientific and ethics review processes meant that risks of serious harm were highly unlikely, the levels of motivation to join HIS seen could raise concerns about study payments being too high. Particular value was placed on the reliability, rather than level, of study payment in this setting, where subsistence livelihoods are common. Study volunteers were generally clear about the study aims at the point of recruitment, and this knowledge was retained over a year later, although most reported experiencing more burdens than anticipated at enrolment. Strict study screening procedures, regular clinical and laboratory monitoring of volunteers, with prompt treatment with antimalarial at predetermined endpoints suggested that the risks of serious harm were highly unlikely. Ethical concerns emerged in relation to volunteers’ attempts to conceal symptoms, hoping to prolong residency periods and increase study payments; and volunteers making decisions that compromised important family relationships and personal values. Our findings support an interpretation that, although study volunteers were keen to join the study to access cash payments, they also paid attention to other features of the study and the general clinical research landscape, including levels of risk associated with study participation. Overall, our analysis shows that the ethical concerns emerging from the study payments can be addressed through practical measures, hinged on reducing burdens and strengthening communication, raising important issues for research policy and planning.

**Supplementary Information:**

The online version contains supplementary material available at 10.1186/s12910-022-00783-y.

## Introduction

In recent years, Human Infection Studies (HIS) have emerged as an important research approach with the potential to fast-track the development of vaccines and treatments for infectious diseases globally. HIS involve deliberately infecting healthy volunteers with a disease-causing pathogen in a controlled environment with the aim of understanding disease pathogenesis or testing vaccine or drug candidates [1]. HIS may provide an early indication of effectiveness of drug or vaccine candidates, underlining important social and economic value, but also raise concerns about a range of potential ethical issues. Core issues concern the acceptability of infecting healthy volunteers with disease causing pathogens, volunteers’ understanding of the concept of deliberate infection, and their experiences of and motivation for participation. Relatedly, there are debates about policy for HIS, including how to support fair decision making processes and compensate for the risks and burdens experienced by volunteers, particularly in low- and middle-income countries (LMICs) [2–7].

In this paper, drawing on social science research embedded within a malaria HIS, we focus on a particular debate in the literature around appropriate levels of study payments for participants in HIS. We use the word ‘payments’ to refer to compensation and re-imbursement, especially when offered in monetary terms. Some features of HIS may prompt higher levels of payment, including prolonged periods of residency and post-discharge study visits often involved as well as physical and other burdens associated with deliberate infection (e.g. restricted movements and regular blood draws for experimental and clinical/safety monitoring) [8–10]. Prolonged residency requirements support science and safety, for example, where repeated, timed blood sampling is needed to support research and a participant’s health must be monitored continuously to diagnose and treat a deliberate infection or other health issues, if these arise [11].

Across the following sections of this paper, we first outline core elements of the debate around the ethics of payments to study participants relevant to this paper, with a focus on research in low resource settings. Following an outline of a programme of malaria HIS conducted at an international collaborative research programme in Kilifi, Kenya, we describe the methods and findings from qualitative research embedded within this programme, involving healthy volunteers and other research stakeholders. In our discussion, we draw on findings from this and earlier publications to assess the nature of ethical concerns around study payments made to malaria HIS volunteers in Kilifi and implications for policy, with potential relevance to other similar settings [7, 8, 12].

### Ethical concerns around payments to HIS participants

A fundamental ethical requirement in any research involving human participants is that there should be a favourable risk–benefit ratio, and that risks of burdens or harms are minimised and benefits maximised [13–15]. In this respect, study benefits—including some forms of payments—should account for costs, burdens and inconveniences linked to research participation and recognise existing structural inequities, including where made as forms of ancillary care [16, 17]. At the same time, offering reasonable support to participants means that benefits should not present an offer that will be ‘empty’ or ‘difficult to refuse’, which may be a particular risk in contexts of unmet need [18, 19]. In contrast, failure to compensate study volunteers adequately risks exploitation at individual and population levels. The latter is illustrated through a phenomenon described as “ethics dumping” in which study benefits and payments are minimised by choosing to conduct studies in low resource settings where low benefits are highly attractive to populations with few alternative sources of cash [20, 21].

The debate around the ethics of payments to study participants maps on to these wider considerations around study benefits, paralleling the need to balance risks of exploitation (at individual and population levels) and ‘undue inducement’ [3, 6, 22, 23]. An earlier definition identified four criteria for undue inducement: that a desirable offer is made, the offer is ‘excessive or irresistible’, leads to decisions that would otherwise not be made to participate and the study potentially involves serious burdens or harm [24]. Refining this definition, others have noted that a ‘high’ offer may distort a study participant’s ability to perceive the risks and benefits of participation accurately [25–27]. At the same time, concerns about undue inducement have been described as unnecessary; prior independent expert ethics review will ensure a reasonable balance exists between study risks and benefits before inviting participation, and payments can therefore only act as due, and not undue, inducement [24]. Others have noted dangers in an overreliance on the optimal functioning of research ethics committees (RECs), including in undertaking a risk–benefit assessment [27].

Additional markers for study payments being ‘too high’ include: (i) the encouragement of forms of deception by volunteers, including concealment of conditions that might preclude participation; (ii) undermining of public trust [6, 28, 29]; (iii) conflicts emerging within communities and families; and (iv) risks that the relationship between researchers and communities will become commercialised [8, 30, 31]. Where payments are ‘too low’ on the other hand, concerns about exploitation centre on risks of disproportionately attracting economically vulnerable participants who may be willing to take on higher burdens or risks of harm to access even low payments; and the impact of being unable to recruit and retain sufficient numbers of participants in studies of high social value [25, 27].

Several studies have also underlined the need to take careful account of context in assessing the appropriateness of levels and forms of benefits and payments to participant [6, 32, 33]. Thus, what might be judged as undue inducement in some context (for example where there is widespread poverty and vast unmet health needs, and thus research participation can be particularly attractive and cloud out key aspects of a study for those joining), maybe due inducement in other contexts. In this way, the distinction between due and undue payments seems to be a relative assessment open to different interpretations, including by ERCs [34].

Finally, ‘appropriateness’ may be more easily assessed for some forms of payment than others. While reimbursement of financial costs can often reference receipts or standard rates for expenses, reimbursement of economic costs is more challenging, particularly in informal economies. However, minimum wage guidance can be helpful where available [35]. Other forms of payment, including compensation as a form of remuneration for services given or as an incentive to participate, clearly require more context-specific judgement to avoid risks of exploitation and undue inducement [6, 36].

### Malaria HIS at the KEMRI-Wellcome Trust Research Programme in Kenya

The Kenya Medical Institute (KEMRI)-Wellcome Trust Research Programme (KWTRP) is a longstanding multidisciplinary, international collaborative research programme in Kenya with main hubs in Kilifi, on the Kenyan coast, and Nairobi. Over the past 10 years, a programme of malaria HIS has been developed and implemented within the programme, based on extensive early consultation with scientific, medical, regulatory and legal bodies within Kenya and internationally to establish an agreed way forward [37]. In addition, in Kilifi, where much of KWTRP’s clinical, laboratory and epidemiological research is conducted, including the malaria HIS programme that forms the basis for this paper, all research is supported by an established community engagement platform. Engagement activities include outreach, open day, interactive and consultation activities with a range of community stakeholders, including community representatives and leaders [38, 39].

Following a proof-of-concept malaria HIS involving 28 healthy adult volunteers with low to moderate prior exposure to falciparum malaria at the KEMRI Clinical Research Centre in Nairobi in 2013, a programme of malaria HIS at KWTRP has taken place between August 2016 and February 2018 in Kilifi. Over this time, the malaria HIS programme has involved a total of 161 healthy adult volunteers from two administrative locations in Kilifi County and one location (Ahero) in Kisumu county in Western Kenya, selected to represent areas of varying malaria endemicity (low and medium endemicity in Kilifi and high endemicity in Ahero) [40, 41]. Kilifi and Kisumu counties lie about 843 km apart, with differing sociocultural features reflecting diversity in predominant cultural traditions (Mijikenda ethnic traditions in Kilifi and Luo in Ahero) and economies. Rates of multidimensional poverty have been assessed at 35.5% and 59.9% in Kisumu and Kilifi counties, respectively [42], and Kisumu town is the third largest city in Kenya.^1^ Volunteers from both counties shared similar economic opportunities and challenges in life, with a majority dependent on subsistence or small scale farming, sea or lake fishing, tourism (in Kilifi) and small scale trade or business, including driving motorbike taxis and selling vegetables, cooked food or household items [43].

The processes involved in the malaria HIS in Kilifi have been described in detail elsewhere [11]. In brief, a dedicated community engagement team in Kilifi and Ahero visited communities to introduce the study to potential volunteers, following which experienced research staff provided additional information about the study to interested individuals, who were screened for eligibility. Following selection and recruitment, including individual informed consent processes with a quiz to check understanding, research clinicians gave an intravenous injection of *P. falciparum* sporozoites to healthy volunteers under closely monitored conditions. Volunteers were required to remain within a residential facility at a university guesthouse in Kilifi town for up to 25 days to support clinical monitoring and fixed-time repeated blood sampling to assess immune responses to the challenge and check for the development of malaria infection. Participants who developed malaria were treated and discharged on full recovery, with variation in the time to develop symptoms, given individual differences in existing levels of immunity. Final clinical follow up for the malaria HIS occurred at day 35 post-challenge, after discharge from the in-patient facility.

In relation to study payment for this HIS, earlier empirical research at KWTRP had supported the development of context-specific guidance on study payments [30, 31]. Given the unique character of HIS in requiring prolonged residency and deliberate infection, the policy on these study payments was developed through a consultative process including the HIS research team, the wider scientific community, and the Community Liaison Group at KWTRP. Payment policies were approved by the relevant ethics and regulatory bodies (KEMRI Science and Ethics Review Unit, and Pharmacy and Poisons Board respectively) [11]. Study payments accounted for reimbursement of travel expenses, and for compensation for financial and economic losses [11]. Travel was reimbursement in relation to actual costs incurred. Compensation and remuneration costs were assessed in relation to likely financial and economic losses, burdens experienced, and burdens accepted during residency, including limitation of movement away from home and experience of malaria infection, where this arose. The total payment, based on these assessments, was set at KSH 2000/day (approximately USD 20/day) drawing on national minimum wage guidance [7] and earlier consultations with KEMRI Community Representatives on daily rates of compensation during their own residential training activities [4, 30, 31]. Since the payments were pegged on days spent at residency (up to a maximum of 25 days), the total amount that individual participants would receive would inevitably differ as it will depend on how long one stayed in residency. The payments were made as a lump sum at the end of residency.

## Study methods

During the malaria HIS in Kilifi, we conducted qualitative research before, during and after the volunteers’ residency period [7, 8, 12]. While observational data were collected during preliminary community engagement activities in Kilifi, the main data collection occurred during HIS residency (Period 1, T1), six weeks after leaving residency (Period 2, T2) and 12–18 months after residency (Period 3, T3). The findings presented in this paper emerged from data collection across all phases. Participants included study volunteers (n = 37), HIS investigators and clinicians (n = 8), community-based frontline staff (n = 27) and community leaders and representatives in Kilifi (n = 25). Table 1 describes participants’ sociodemographic characteristics. All methods were performed in accordance with the relevant guidelines and regulations, including the Declaration of Helsinki.

**Table 1:** Demographic characteristics of study participants

Location	Kilifi		Ahero		Total (%)
Characteristics	Male	Female	Male	Female	
*Volunteers*					
Age (years)					
19–29	3	5	4	3	15 (40.5%)
30–40	6	3	3	3	15 (40.5%)
41–51	2	3	1	1	7 (18.9%)
Education level^a^					
None	0	2	0	0	2 (5.4%)
Primary	5	5	1	1	12 (32.4%)
Secondary	3	1	4	4	12 (32.4%)
Tertiary	0	1	3	2	6 (16.2%)
Unavailable	3	2	0	0	5 (13.5%)
Occupation					
None	3	4	0	1	8 (21.6%)
Student	0	0	3	0	3 (8.1%)
Subsistence farming	1	5	0	0	6 (16.2%)
Self-employed/business	5	1	5	5	16 (43.2%)
Employed	2	1	0	1	4 (10.8%)
Total	11	11	8	7	37 (100%)
*Other stakeholders*					
Category of stakeholder					
Investigators/clinicians	1	4	3	0	8 (13.3%)
FWs/CHVs	12	7	1	3	23 (38.3%)
CLG/CE staff	2	1	–	1	4 (6.7%)
Chiefs	2	1	2	0	5 (8.3%)
KCRs	12	8	0	0	20 (33.3%)
Total	29	21	6	4	60 (100%)
Total participants	40	32	12	11	97

Adapted from Chi et al. [8]

^a^Information on occupation for 5 volunteers is missing

### Sampling and data collection

Sampling processes in this qualitative study were generally purposive, based on maximising diversity around characteristics or experiences likely to be important to our analysis [44]. However, at T1 all HIS volunteers were invited to participate in the study due to the small study population. During T2, sampling aimed to capture diverse HIS experiences, while at T3 diversity in gender, home location, time of diagnosis and duration of inpatient stay were considered. Study staff were sampled based on their roles (clinician, study investigator, field worker/community health volunteer and community engagement staff), and community leaders (from Ahero and Kilifi) and community representatives (from Kilifi) were invited based on residency within the locations involved in the HIS. The community representatives from Kilifi known as the KEMRI Community Representatives (KCRs) is a hybrid network of community members elected by their community to serve for a period of time as a hybrid community advisory board [38]. Data collection methods included non-participant observation, individual and pairs in-depth interviews and focus group discussions, which are described in detail elsewhere [8, 12] and summarised in Table 2 below. Tools used for data collection are included in Additional file 1.

**Table 2:** Data collection methods

Participants	T1 Kilifi	T2 Kilifi	T3 Kilifi and Ahero
	During residency	6 weeks after residency	12–18 months after residency
	Data collection method/number participants	Data collection method/number participants	Data collection method/number participants
Community engagement staff and community members	Observations during community engagement		IDIs in Kilifi and Ahero; n = 4; 30–90 min; At KWTRP offices/ACTU
HIS volunteers	Observations in residential facility; FGDs with HIS participants from Kilifi; n = 32; Duration: 80–180 min; In residence	IDIs with HIS participants from Kilifi n = 5 (2 participated in FGDs at T1); 30–90 min; At nearest local dispensary/Community	IDIs/pairs interviews with Kilifi and Ahero HIS participants; n = 18; 30–90 min; At nearest local dispensary
Study clinical staff	IDIs; n = 3; Duration: 30–90 Min; In residence		IDIs in Kilifi and Ahero; n = 5; 30–90 min; KWTRP offices/ACTU
Front line staff/community-based	FGDs; n = 14; Duration: 80–180 min; At local dispensaries in community		IDIs in Kilifi and Ahero; n = 12; 30–90 min; At KWTRP offices/ACTU
Community leaders and representatives	FGDs at local dispensaries in community; n = 20; Duration: 80–180 min		IDIs in Kilifi and Ahero; n = 5; 30–90 min; At KWTRP offices/ACTU

### Data management and analysis

All individual IDIs, PIs and FGDs were audio recorded, transcribed verbatim and translated into English where needed, a process that was quality checked by two members of the team. We imported data into QSR Nvivo 12 software to support our analysis, using a Framework Analysis approach [45]. Framework analysis involves steps of close reading of transcripts and field notes, the selection of three rich accounts to inform the development of a preliminary coding scheme by consensus within the study group and coding of all transcripts as an iterative process in which the scheme was adapted to capture emerging findings. Saturation was attained when no relevant new codes were being identified around the broad topic of interest (i.e., payment issues) from the transcripts. The team discussed data within codes to support organisation into themes, with findings charted into a matrix by individual participant or group, to support further interpretation. This overall process of analysis and interpretation informed earlier publications [8, 12], based on data collected during T1 and T2. In this paper, we present and analyse data from T1, 2 and 3, to focus on an account of ethical concerns around study payments in the Kilifi malaria HIS, drawing on the wider literature including that presented in Sect. 1.1.

As highlighted in our earlier publication [12], throughout the tool development, data collection and analysis, we were conscious of and made every effort to consider our positionality, especially as staff/researchers from an institution (KWTRP) perceived as highly prestigious and powerful across the study communities as well as our individual views about HIS. The study team included both social scientists (PCC, VM, IJ, EO, DK) and basic scientists (MK, PB), with extensive experience of community-based clinical research, including within the study communities. Additionally, some members of the social science team (IJ, DK) were introduced to study volunteers during screening, enrolment and study engagement activities, giving them an opportunity to build rapport prior to data collection. Taking these into considerations, we presented our social science study as a separate study from the clinical study, with separate recruitment and consenting processes. We were therefore keen to maintain a neutral stance on HIS throughout the data collection and analysis process.

### Ethical considerations for the qualitative study

Ethics approval for this study was sought from Kenya Medical Research Institute (KEMRI) Scientific and Ethics Review Unit (SERU No: KEMRI/SERU/CGMRC/029/3190 & 147/3808) and Oxford Tropical Research Ethics Committee (OxTREC Reference No: 2-16 and 16-19. Prior written informed consent was sought from all study participants.

## Findings and discussion

Across this section, we present and discuss findings that support consideration of ethical issues around study payments during the malaria HIS, as outlined in the introduction to this paper. Drawing on the literature, we consider the extent to which payments may have influenced individual and population level motivation to join the study, could have led to important values being undermined, deceptions practised, or volunteers’ judgement being ‘clouded’ in assessing the balance of benefits and burdens involved in participation. We also consider an influence from volunteers’ level of understanding of the study at the point of deciding to join and identify policy implications for these findings.

### A Population and individual level motivation to join the study: An influence from study payments

Across our data, the study payment emerged as one of the main reasons that most people were keen to join the malaria HIS, meeting the definition of a ‘desirable’ good. Even a year later, most volunteers reported a high level of satisfaction with the study payment given, with the reliability of this income a particularly important feature:

I was satisfied, I was grateful, because even if I would have stayed at home for the one month where would I have gotten the money from? I wouldn’t have got it, what kind of work would I have done to get that money, I wouldn’t get, so according to me, I was grateful… (P01, F, KF, T3)

The cash payments were not, however, the only reason that volunteers were motived to join the study. Other reasons included a strong desire to contribute towards the development of new malaria vaccines to support local communities and accessing screening health checks without cost. Perhaps more importantly, volunteers described feeling reassured about the safety of the study procedures, including through expressions of trust in the research institution and protocol review process, hearing positive accounts of the study from participants in earlier phases of the study, interactions with study staff and a general perception of malaria infection as an easily treated, everyday condition [7, 8, 12].

#### A high interest in joining the HIS

One potential reflection of the desirability of the study payment per se was the unusually high level of interest in participating in the study shown during community engagement activities where the study staff introduced the study in rural locations in Kilifi and Ahero. Earlier phases of the malaria HIS programme faced early challenges with initial recruitment – which was attributed to unfamiliarity of this type of study design, concerns around safety and waiting to see whether those who sign up first will be safe. The amounts of compensation were the same across all the malaria HIS. However, in the later phases of the malaria HIS frontline staff and KEMRI Community Representatives noted a much greater interest amongst local residents in joining the current phase of the study. Levels of volunteering were also far more than that seen in earlier (non-HIS) trials in Kilifi, such as vaccine and drug trials [8], where the levels of inconveniences are much lower. Further, our observations during screening periods to determine eligibility to join the study suggested that many potential volunteers experienced high levels of anxiety while waiting, and others showed marked disappointment on learning they would not be able to take part. Some volunteers found to be eligible during initial health checks also worked hard to ensure they ‘made it’ into the final study cohort, including take measures to minimise risks of getting malaria before the challenge event that would prevent their final inclusion:

I had to stay here at home under guard…under a mosquito net…to prevent myself from getting malaria again…because I heard that when you go on the last day and … [and] you are found with malaria, then you come back home. (P03, M, KF, T3)

Similarly, as we describe in detail elsewhere, tensions emerged at times between community-based frontline HIS staff and community members who had not been offered an option to be screened for eligibility, leading to complaints of bias [8]. This strong interest in joining the study was shown in a local newspaper article that was reporting the first social science study undertaken in the initial phases titled: “Want cash? Volunteer for a dose of malaria parasite, says KEMRI amid ethical queries” [46]. The article seemed to increase public interest in participating in the HIS and prompted a response from KWTRP and KEMRI headquarters to address the issues raised [47].

#### A high interest in maximising payments

A second clear reflection of the influence of the study payment was linked to the payment approach, given as a lump sum given at the end of residency and tied to the number of days spent, as an economic cost. This was done for practical reasons as it was easier for the institution to disburse payments once instead of more regularly. However, volunteers knew that the longer in-residency one stayed, the higher the amount one would eventually take home, as stated by a respondent:

So, there was a sense in which someone knew what s/he will get after that research [cash compensation], so even if you will not pray loudly for people to hear …you will pray and say these parasites should not result into malaria until 21 days are over, but I didn’t last for the 21 days (P05, M, KF, T3)

While it would not have been possible for participants to conceal symptoms given frequent clinical and blood monitoring, within one cohort, a competition seemed to emerge among HIS volunteers to ‘make it to the end’, suggesting a form of collusion to support hiding or delay in reporting symptoms:

My colleagues were telling me “persevere, there are only a few days remaining…don’t report [symptoms]… because you will be given medication and you’ll go home and it’s still not yet time” but I told them “no I can’t persevere I must report this so that I’m given medicine.” (P02, F, KLF, T3)

### Competing values on participants’ decisions about the study

For health research in low resource settings in general, a proposed marker for payments being ‘too high’ is that participants may trade-off personal values against financial gain [29, 33, 48]. From our data, it emerged that individuals hoping to join the malaria HIS faced many challenges in setting up arrangements that would allow them to be away from home in residency for up to 25 days. There were particular worries and practical concerns for some mothers leaving young children behind, and for individuals who were the main family ‘breadwinners’ since payments were only made as a lump sum at the end of the study. The anxiety around the welfare of their families at home reported by some volunteers during residency [8] underlines the high value attached to the payments.

Volunteers and study staff also described instances where eligible individuals were unable to enroll in the study because of disagreements within families. Participation caused a family rift between one volunteer and his sister when he decided to join the HIS, and gave up responsibility for managing his sister’s hotel:

She said you just go [to join the HIS], but if you go, we’ll never be on good terms again… So, from that date up to today, she has never called me again. Even when I call her, she doesn’t receive my calls. (FGD5, M, AH, T1)

Mandatory use of effective contraceptive for female study participants of reproductive age enrolled in research is common to most clinical trials [49, 50], as was the case for the HIS study. However, some women volunteers joined the HIS despite unwillingness to comply with the requirement to use an effective method of contraception, which aimed to limit maternal and foetal risks associated with clinical malaria. Some were outrightly opposed to the use of contraceptives or felt unable to follow instructions for health reasons. Others said they were unaware of this requirement. Overall, for this group, a sense of ‘being forced’ to do something unwanted emerged, showing potential to undermine their values.

…it so happened that even that part of Femiplan [contraceptive method] slipped me off [did not register]. So I came all the way here [from Ahero] not even having an idea about that…they asked me about family planning and I said I’m not using any. So it became a situation whereby I had no choice, I have to use it… (FGD4, F, AH, T1)

…she qualified but didn’t want any kind of [contraceptive]… she went, then she came back…you find that she is taking this [contraceptive] not because she really wants but because she wants the study…She tried to get into the study without the [contraceptive] and we could not accept, yeah. (Study Staff 01, F, KF, T3).

### Unanticipated burdens and on-going challenge of managing expectations

An approach that has been proposed and is now widely practiced is to check for volunteers of the study that they are recruited to, prior to enrollment. This is especially the case for clinical trials where significant information is provided and the participants need to understand these, particularly the risks and burdens of participation. A number of tools have been used, and are publicly available to researchers to use in these assessments. As was reported elsewhere [7, 11], during the consent process, prospective volunteers were required to pass a quiz^2^ assessing cognitive understanding of core elements of the study, including aims and procedures [7]. In addition, community-based frontline staff and volunteers in earlier HIS studies were often asked questions about the study and some shared their experiences with friends and neighbours. It is also recognized that undue inducement can occur where high study payments ‘cloud’ study participants comprehensive appreciation of key information about the study particularly the risks and burdens leading to ‘poor’ judgement [51–53].

#### Strengthening understanding at the point of enrollment

From our findings, study volunteers were generally clear about the study aims at the point of recruitment, and this knowledge was retained over a year later. Given the restricted scope and multiple-choice nature of the quiz used during consent, the depth of understanding of study procedures, including risks of physical harms (malaria infection) and burdens (the challenge event and subsequent venous blood sampling), was more difficult to establish at the point of recruitment. A range of unanticipated psychosocial harms and burdens emerged for volunteers over the course of the study, unanticipated even by the study team, including anxiety about the nature of the challenge injection (if it might contain a more ‘deadly’ pathogen like HIV or contaminated [7, 12]. Other anxieties concerned the security of their prior livelihoods while in residence and issues around interpersonal conflict in residency [8]. Steps were taken to strengthen some aspects of the informed consent process during the study, including practical demonstrations of the amount and way blood samples would be taken.

In other studies, volunteers in a malaria HIS in the USA, who had a good understanding of the burdens described at the outset, later described these as greater than anticipated [9]. The emergence of ‘unanticipated’ types or levels of psychosocial burdens points to the importance of careful monitoring of volunteers’ experiences in the post ethics approval space, including through embedded social science, and the need for research planning to be responsive to emerging issues throughout its implementation. This form of monitoring and responsiveness seems particularly important for studies based on novel approaches, allowing future study planning, community engagement and informed consent processes to be adapted to take account of stakeholders’ experiences.

#### Managing expectations, a longer-term view

To make an assessment of the practical value of study payments over time, we discussed volunteers’ attitudes to and use of payments with volunteers a year after their participation. Table 3 summarises former volunteers’ accounts of the ways in which the compensation was used.

**Table 3:** Volunteers’ reported use of study payments at T3

ID	Gender M/F	Reported use of compensation/economic status
P06_AH	F	Invested in small-scale business; gave part to sister for college fees
P08_AH	F	Used most on mother’s funeral expenses; bought additional stock for small business; paid *“merry go round”* (savings group) arrears
P10_AH	M	Invested in horticulture farming and used the proceeds to tile his house floor
P12_AH	M	Completed a driving course, begun before joining the study; bought food for family on way home; helped parents to buy house window grills; bought some sheep and goats; and invested in small shop
P13_AH	F	Bought household utensils and a cupboard for parents; replaced lost certificates needed for a future college application (funds insufficient at present); started a small shop
P14_AH	F	Invested in own small-scale business; shared cash with close family members
P15_AH	M	As a college student, used payment on accommodation and tuition fees
P16_AH	M	Returned to college course earlier suspended through lack of fees; shared cash with close family members; other personal use
P17_AH	F	Shared cash with parents and sister, who had provided childcare over study period; paid college fee debts needed to access certificate
P18_AH	F	Payment mainly used on health care for children, who were unwell on her return; settled outstanding rent payments; and made payment to person providing childcare while in residence
P01_KF	F	Paid school fees; bought some goats
P02_KF	F	Paid for children’s school fees and school items (books, shoes, bags)
P03_KF	M	Bought a cow and set up business selling milk/tapping for palm wine at home, where he could also farm. Cash enabled him to relocate back home from a nearby urban centre, where he had been resident and selling fried food
P08_KF	F	Expanded small business selling vegetables, buns and fried potatoes; enrolled her child in a school of her preference
P05_KF	M	Paid children’s school fees; invested in their small farm
P06_KF	M	Repaired house roof; wife invested in her small shop; supported daily living costs
P07_KF	F	Bought a cow to generate income through selling milk and breeding
P04_KF	F	Paid children’s school fees; bought a cow as small business investment

While some found this a difficult topic to discuss, hinting that they had not, and did not, feel free to say what payments should be, most described a reasonable level of satisfaction with the amount provided:

…to say the truth, I can’t say it was enough or not because you know you can be employed, and the boss says I will pay you this much, will you need more? And s/he has already said that, and you have been told its voluntary and withdrawing also is voluntary, so what will you object there, so whether it’s enough or not… (P03, M, KF, T3).

On my side…according to what I get from the market I thought it was ok (P06, F, AH, T3)

As shown on Table 3, nearly all volunteers had been able to make positive changes in their lives using the cash. But since cash had often been used to address pre-existing challenges, for example, paying off debts for school or college fees, certificates and dowries, they had not experienced an important change in their living conditions or livelihoods but returned to the same often challenging economic situations. For some, earlier optimism about the extent to which the study compensation was likely to change their lives felt misplaced.

Aaah, my economic status as for now, it’s not that impressive because it has become difficult … because the little that I get most of the time goes to school fees, because I have five children… (P05, M, KF, T3)

A few volunteers felt that the study payment had not adequately accounted for the costs and burdens involved in participation, and strongly recommended an increase in the rate in future similar studies, at least in line with inflation:

Actually, we were told it was not like they were paying us…they said they were compensating us, to appreciate us for our time, because we took time to go to Kilifi, to be taken blood, you know literally if you …were to pay somebody, you will pay much more…but they said they were only going to compensate us for our time (P14, F, AH, T3)

In a few cases, individuals mainly used the cash payments to cover costs incurred while they were in residency and seemed therefore not to have benefitted to any great extent:

I kept using the money for his treatment [a child], my daughter was also having some pain in her stomach. So the money didn’t help me that much because …you know I am a single parent so… I also gave someone who used to look after the children two hundred shillings daily (2USD), so when I came [home] I also paid for that (P18, F, AH, T3)

Most volunteers used the cash to make modest but important investments in businesses and buildings, including homes (for example, renovating homes or business premises). Many also shared the cash within families to support a range of smaller activities.

After returning from there [study residency] is when I expanded the business and it is now bigger …when I left there I had money, then I increased my stock that’s when it became bigger and my profit as well. (P08, F, KF, T3)

Similarly, a few individuals invested study payments in ways likely to bring positive changes to their lives in future (for example, through investments in livestock):

Ah! I got money and bought a cow, I now hope that life will be fine, because when it delivers, I will be [a step ahead]. (P07, F, KF, T3)

The accounts of community leaders and study staff largely supports this account of a range of outcomes, suggesting that most participants benefitted in relatively important, but arguably not transformational ways, including buying livestock and second-hand motorbikes, investing in businesses, and repairing homes. Rarely described were instances where the cash was used in ways that were unhelpful, including in buying recreational drugs and alcohol. Another factor that emerged was the gendered sociocultural challenges in relation to bringing cash into the family [8]. Community leaders and frontline community-based staff described instances in which family conflicts emerged over the payments. For example, some women volunteers were unwilling to hand over cash to male household head, as would be expected in this traditionally patrilineal culture, and sometimes serious disagreements arose about how the cash should be used [30, 54]. While these issues are arguably outside the responsibility of researchers, family conflicts are important considerations in this analysis.

### Reflections over time and willingness to join HIS in future

Across this data, a number of former volunteers reflected on their earlier *misplaced* optimism about the extent to which the study compensation was likely to change their lives. Even then, most described a willingness to join another similar study in the future, a position held during the time of their participation, a few weeks after they left the study and 12–18 months later [8, 12]. This generally positive view about participation was tied to the overall experience of participating in the study, including the care with which they were taken, the value of the cash compensation, enjoyment of the facilities provided (having a good rest), being able to contribute towards developing a malaria vaccine, the opportunity to travel to a new place (for Ahero participants) and the fact that good clinical monitoring was available during residency [8]. Several mentioned they would recommend joining a study like this to family or friends:

I would do it again, it was a great experience anyway yeah, at some point we got used to it. What I was so afraid of was the blood draws but first, second, third day I just got used to it and it was just ok. I enjoyed, that’s why I am telling you if given another chance I would go for another one. (P17, F, AH, T3)

Some participants who were unemployed or had unstable jobs or source of income were particularly interested in joining future HIS, seeing this as reliable^3^ (although small) source of income:

The motivation of participating again is you know here we are jobless, so if that happens again, there is that small reimbursement when you succeed so you get that and when you get that you add on what you have, I think that is good, and that’s why I have said when that project comes again, then I should be one of them…at least I move up a bit (P03, M, KF, T3)

Of two volunteers who described unwillingness to join an HIS in future, their reasons were not related to the amount of compensation but to challenges experienced during participation, including the experience of malaria symptoms and conflicts with other volunteers during residency [12]:

I think my blood doesn’t want any strange thing… I mean with my blood I think I cannot participate… mean I have been thinking like that because they were many who remained okay but as for me, I got problems. So maybe my blood doesn’t want any other thing (P05, F, KF, T2)

### Assessing ethical concerns around levels of study payments

Overall, our findings support an interpretation that, although participants were keen to join the study to access cash payments, they also paid attention to other features of the study and the general clinical research landscape, including levels of risk associated with study participation, as has been observed among healthy volunteers participating in HIS [10, 55] and Phase I clinical trials [56, 57]. In this way, while accessing payments was a main motivating factor for joining the study, this assessment was based on positive support for the study aims, given local relevance, and reassurance about safety through existing trust relations with KWTRP and its staff, familiarity with the nature and treatment of malaria as an illness, and some recognition of the regulatory processes that protect volunteers’ interests. These accounts underline that careful thought processes often went into decision making about participation, supporting an idea of appropriate judgment.

#### Were the study payments ‘too high’?

To address this question, our point of departure is the recognition that making a determination whether the study payments were ‘too high’ is a challenging exercise. First, drawing on the markers for undue inducement explored in Sect. 1.1, these seem not to suggest that the payments were ‘too high’ to unduly influence participation in the study, but it also emerges that they may not cover all the bases of what might influence people to participate. Other factors however do point to the fact that some participants placed greater emphasis on the payment offered in the study in terms of compensation and re-imbursement – particularly the monetary lumpsum offered at the end of the residency period. These seems to have influenced some choices that were made.

However, from our findings, the main consideration that the study payments may have been ‘relatively high’ comes from the unusually high levels of interest in participation from community members in both Kilifi and Ahero. This is particularly clear in Kilifi, the site of the longstanding research programme, where these levels of interest have not been seen in the past for clinical studies, including those aiming to impact burdens of malaria. The emergence of conflict between community-based frontline research staff and community members, related to accusations of partiality or unfairness in volunteers’ recruitment, and the reported impacts on community attitudes towards joining other types of studies as reported in our earlier study [8], also speak to levels of interest that may be high. Similarly, some volunteers’ attempts at concealing symptoms during the trial (even though not feasible in practice) and reports of ‘trading off’ important existing values against decisions to join studies (where the primary motivation to join was financial) raise similar concerns, as reported for other non HIS clinical research [58, 59]. Concealment or delay in reporting data further raises concerns around volunteers’ safety, research integrity and judicious use of research resources [28, 60].

Additionally, we have argued that there were important unanticipated psychosocial burdens experienced during the conduct of the study. These did not lead to volunteers terminating their participation. Recognising that some of these unanticipated burdens become clear later, consent should thus be a process with on-going checking of whether volunteers are still accepting to continue participating and should be more responsive to context, potentially requiring on-going monitoring and feedback loops.

Finally, our previous studies, and studies elsewhere have shown that, while ‘too high’ payments raise concerns around undue inducement, low payments can be exploitative. This re-emphasizes the dilemma that is faced with payments; offer too little and is exploitative, offer too much and it is undue inducement. The fair offer arguments provide a balance, taking account of contextual considerations and what can be a fair offer for participation in different types of research, recognizing that what might be fair in one context might be considered different in other contexts, and over time.

#### Countering concerns that payments were ‘too high’

Three core considerations counter concerns about payments being ‘too high’. Firstly, the high levels of interest in joining the study seem to have been underpinned by often unrealistic expectations of how the payments would impact volunteers’ lives. One response might therefore be to strengthen volunteers’ capacity to make this assessment, for example, by involving previous HIS volunteers in engagement activities, rather than reduce the payments. Secondly, changes in the way payments were made, rather than the amount given, for example regular weekly payments to volunteers during residency rather than a lump sum at the end [8]. Additionally, former volunteers’ reports on the way that payments were used illustrate great diversity in the extent to which individuals have been able to make important changes to their lives, suggesting that these levels are reasonable.

If not ‘too high’, we should consider whether the payments made were ‘too low’. However, many of the findings above suggest this is not the case. An additional point is that, in spite of the burdens experienced, no former volunteers expressed regret about their decision to participate and the vast majority said they would be happy to join another similar study in future, or recommend this to a family member or friend [8, 12]. Study participants’ regrets about their decision to participate in a study has been identified as an important marker of undue inducement [61, 62]. As one former volunteer appropriately recommended, payments should however take account of levels of inflation over time.

#### Policy implications

Based on this research, the findings show that payments made to volunteers in the Kilifi malaria HIS align with important ethical principles around payments in general. However, there are a number of important policy recommendations that can be made to underpin ethical practice around payments in this and other similar settings. These include the need to: (i) Build a more grounded community and individual understanding of malaria HIS to strengthen informed consent and reduce risks that psychosocial burdens are unexpected, including through the involvement of former HIS volunteers in designing and implementing engagement activities; (ii) Replace lump sum payments with weekly payments, to avoid creating a spuriously high sense of their value and reduce burdens for families at home over this period; (iii) Ensure greater transparency and fairness in recruitment policies across communities; and (iv) Optimise volunteers’ experience of residency, for example, by providing access to skilled psychosocial support, offering life-skills capacity-strengthening opportunities, allowing more flexibility of movement, and supporting greater family contact during residency, in alignment with earlier recommendations for maximizing perceived benefits and minimizing perceived burdens of study participation [8].

Additionally, it is important to recognize that motivation to join HIS and other clinical studies without the prospects of any therapeutic benefits such as phase I clinical trials, go far beyond the size of study payments. As highlighted in our earlier study, volunteers’ perceptions of what constitutes a study benefit differs from what study teams and ethics and regulatory authorities’ typical characterization of ‘study benefits’, and include non-financial incentives such as access to free clinical assessments, comfortable living conditions during residency, and opportunities for learning new things and making new friends, among others [8]. This suggests that any appropriate assessment of concerns around undue inducements should equally include non-financial incentives.

A final caveat is to recognise the importance of including a wider set of voices in the analysis we present, through the inclusion of community and HIS volunteer voices in identifying and weighing up the ethical considerations discussed in this paper. While expert ethics review bodies have been recognised as core in making judgements around the acceptability of study payments in HIS, engagement with local health and community stakeholders to support this assessment is also recognised as important [4, 30, 63]. On this basis, in future work, we plan to involve community members in Kilifi in deliberative forms of discussion around the ethics of HIS, including the issue of what constitutes appropriate levels of payment for volunteers, with comparisons between high-income and low-income settings, and across low-resource settings.

## Conclusion

Payment of HIS volunteers is a controversial issue, especially in the context of the ongoing COVID-19 pandemic, with the main ethical issue being concerns about undue inducement and to a lesser extent, the risk of exploitation. In this empirical ethics study, we have explored the ethical issues with volunteers’ payments in a malaria HIS at a low resource setting in Kenya. While the payments to volunteers as reimbursement and compensation were recognized as a desirable good, with exceptional high levels of interest to participate in the study across the study communities, volunteers understanding of the study aims and key elements of the study procedures, do not suggest that volunteers had ‘clouded’ judgement about the study. Moreover, documentation of the way the payments were used by volunteers show that while the payments were highly valued, it was arguably not transformational to be deemed as an excessive offer. Additionally, the design of the study involving close and regular clinical and laboratory monitoring of volunteers after challenge while in residence, with prompt treatment of those that developed clinical malaria ensured volunteers safety and substantially reduced the risk of occurrence of serious harm. Overall, with appropriate national and local ethics and regulatory systems, and study design, concerns about undue inducement or influence in HIS, especially in low resource settings could be addressed. Embedded social science studies can play a critical role in identifying important social and ethical issues prior, during and after the implementation of HIS and other clinical studies and develop appropriate strategies to address them.

## Supplementary Information

**Additional file 1.** Data collection tools.

## Data Availability

The datasets generated during and analyzed during the current study are not publicly available due to ongoing analysis and anonymisation of the datasets but are available from the corresponding author on reasonable request.

## Footnotes

1 https://www.mistraurbanfutures.org/en/lip/kisumu.

2 The quiz is available at: https://wellcomeopenresearch.s3.amazonaws.com/supplementary/14439/1e9075ea-ae3d-4e75-bc90-1418abcae614.pdf

3 Reliability was based on the fact that volunteers were confident that their compensation and reimbursement will be disbursed after participation which is not always the case with some routine casual work they undertake.

## References

[1] Gordon SB (2017). A framework for controlled human infection model (CHIM) studies in Malawi: report of a wellcome trust workshop on CHIM in low income countries held in Blantyre, Malawi. Wellcome Open Res.

[2] Gbesemete D (2020). Exploring the acceptability of controlled human infection with SARSCoV2—a public consultation. BMC Med.

[3] Grimwade O (2020). Payment in challenge studies: ethics, attitudes and a new payment for risk model. J Med Ethics.

[4] Jao I (2015). Research stakeholders’ views on benefits and challenges for public health research data sharing in Kenya: the importance of trust and social relations. PLoS ONE.

[5] Kapumba BM (2020). Stakeholder views on the acceptability of Human Infection Studies in Malawi. BMC Med Ethics.

[6] Lynch HF (2021). Promoting ethical payment in human infection challenge studies. Am J Bioeth.

[7] Njue M (2018). Ethical considerations in Controlled Human Malaria Infection studies in low resource settings: experiences and perceptions of study participants in a malaria challenge study in Kenya. Wellcome Open Res.

[8] Chi PC (2021). Understanding the benefits and burdens associated with a malaria human infection study in Kenya: experiences of study volunteers and other stakeholders. Trials.

[9] Kraft SA (2019). Exploring ethical concerns about human challenge studies: a qualitative study of controlled human malaria infection study participants’ motivations and attitudes. J Empir Res Hum Res Ethics.

[10] Toto NM (2021). “At first, I was very afraid”—a qualitative description of participants’ views and experiences in the first Human Infection Study in Malawi. Wellcome Open Res.

[11] Kapulu MC (2019). Controlled Human Malaria Infection in Semi-Immune Kenyan Adults (CHMI-SIKA): a study protocol to investigate in vivo Plasmodium falciparum malaria parasite growth in the context of pre-existing immunity. Wellcome Open Res.

[12] Jao I (2020). Deliberately infecting healthy volunteers with malaria parasites: Perceptions and experiences of participants and other stakeholders in a Kenyan-based malaria infection study. Bioethics.

[13] Council for International Organizations of Medical Sciences (2016). International ethical guidelines for health-related research involving humans.

[14] Emanuel EJ (2004). What makes clinical research in developing countries ethical? The benchmarks of ethical research. J Infect Dis.

[15] National Commission for the Protection of Human Subjects of Biomedical and Behavioral Research, The Belmont report: ethical principles and guidelines for the protection of human subjects of research. Vol. 2. 1979: National Commission for the Protection of Human Subjects of Biomedical and Behavioral Research.

[16] Hyder AA, Merritt MW (2009). Ancillary care for public health research in developing countries. JAMA.

[17] Richardson HS, Belsky L (2004). The ancillary-care responsibilities of medical researchers: an ethical framework for thinking about the clinical care that researchers owe their subjects. Hast Cent Rep.

[18] Benatar SR, Singer PA (2010). Responsibilities in international research: a new look revisited. J Med Ethics.

[19] Kingori P (2015). The ‘empty choice’: a sociological examination of choosing medical research participation in resource-limited sub-Saharan Africa. Curr Sociol.

[20] Saleh S (2020). Participant compensation in global health research: a case study. Int Health.

[21] Schroeder D, et al. Ethics dumping: case studies from north-south research collaborations. Springer; 2018.

[22] Ballantyne A (2008). Benefits to research subjects in international trials: do they reduce exploitation or increase undue inducement?. Dev World Bioeth.

[23] Largent EA (2012). Money, coercion, and undue inducement: a survey of attitudes about payments to research participants. IRB.

[24] Emanuel EJ, Currie XE, Herman A (2005). Undue inducement in clinical research in developing countries: Is it a worry?. The Lancet.

[25] Largent EA, Lynch HF (2017). Paying research participants: the outsized influence of “undue influence”. IRB.

[26] Lee E (2019). Our flawed approach to undue inducement in medical research. Bioethics.

[27] Resnik DB (2015). Bioethical issues in providing financial incentives to research participants. Medicolegal Bioethics.

[28] Mngadi KT (2017). Undue inducement: a case study in CAPRISA 008. J Med Ethics.

[29] Williams EP, Walter JK (2015). When does the amount we pay research participants become “undue influence”?. AMA J Ethics.

[30] Njue M (2014). What are fair study benefits in international health research? Consulting community members in Kenya. PLoS ONE.

[31] Njue M (2015). Benefits in cash or in kind? A community consultation on types of benefits in health research on the Kenyan Coast. PLoS ONE.

[32] Chi PC (2021). Considering the importance of context for ethical practice on reimbursement, compensation and incentives for volunteers in human infection controlled studies. Am J Bioeth.

[33] Ndebele P, Hyder AA (2021). Promoting ethical payments in human challenge studies conducted in LMICs: Are we asking the right questions?. Am J Bioeth.

[34] Klitzman R (2013). How IRBs view and make decisions about coercion and undue influence. J Med Ethics.

[35] Dickert N, Grady C. What's the price of a research subject? Approaches to payment for research participation. Mass Medical Soc; 1999. 10.1056/NEJM199907153410312 10403861

[36] Różyńska J (2021). Research participants should be rewarded rather than “compensated for time and burdens”. Am J Bioeth.

[37] Hodgson SH (2015). Lessons learnt from the first controlled human malaria infection study conducted in Nairobi, Kenya. Malar J.

[38] Kamuya DM (2013). Engaging communities to strengthen research ethics in low-income settings: selection and perceptions of members of a network of representatives in Coastal Kenya. Dev World Bioeth.

[39] Marsh V (2008). Beginning community engagement at a busy biomedical research programme: experiences from the KEMRI CGMRC-Wellcome Trust Research Programme, Kilifi, Kenya. Soc Sci Med.

[40] Degefa T (2017). Indoor and outdoor malaria vector surveillance in western Kenya: implications for better understanding of residual transmission. Malar J.

[41] Mogeni P (2016). Age, spatial, and temporal variations in hospital admissions with malaria in Kilifi County, Kenya: a 25-year longitudinal observational study. PLoS Med.

[42] Kenya National Bureau of Statistics, Comprehensive Poverty Report 2020. Children, youth, women, men & the elderly: from national to county level. 2020, Kenya National Bureau of Statistics: Online.

[43] Kenya national Bureau of statistics, Basic report on well-being in Kenya: based on the 2015/16 Kenya Integrated Household Budget Survey (KIHBS). 2018, Kenya national Bureau of statistics: Online.

[44] Patton MQ. Qualitative research & evaluation methods: integrating theory and practice. Sage; 2014.

[45] Gale NK (2013). Using the framework method for the analysis of qualitative data in multi-disciplinary health research. BMC Med Res Methodol.

[46] Gathura G. Want cash? Volunteer for a dose of malaria parasite, says Kemri amid ethical queries. 2018 [cited 2020 Dec 12]. https://www.standardmedia.co.ke/health/article/2001283428/want-cash-volunteer-for-a-dose-of-malaria-parasite-says-kemri.

[47] KEMRI. Response to an article carried in the standard titled: “Want Cash? Volunteer for a dose of malaria parasite, says Kemri amid ethical queries” by Gatonye Gathura. 2018 [cited 2020 Dec 15]. https://www.kemri.org/wp-content/uploads/2019/11/RESPONSE-TO-AN-ARTICLE-CARRIED-IN-THE-STANDARD-ON-MALARIA-TRIALS.pdf.

[48] Dickert N, Grady C. Incentives for research participants. The Oxford textbook of clinical research ethics, 2008: p. 386–396.

[49] Schonfeld TL (2009). Women and contraception in research: a pilot study. J Womens Health.

[50] Sullivan KA (2019). Women’s views about contraception requirements for biomedical research participation. PLoS ONE.

[51] Arnason G, Van Niekerk A (2009). Undue fear of inducements in research in developing countries. Camb Q Healthc Ethics.

[52] Emanuel EJ (2005). Undue inducement: nonsense on stilts?. Am J Bioeth.

[53] Savulescu J (2001). The fiction of" undue inducement": why researchers should be allowed to pay participants any amount of money for any reasonable research project. Am J Bioeth.

[54] Molyneux CS (2002). Intra-household relations and treatment decision-making for childhood illness: a Kenyan case study. J Biosoc Sci.

[55] Hoogerwerf M-A, de Vries M, Roestenberg M (2020). Money-oriented risk-takers or deliberate decision-makers: a cross-sectional survey study of participants in controlled human infection trials. BMJ Open.

[56] Fisher JA (2018). Healthy volunteers' perceptions of risk in US Phase I clinical trials: a mixed-methods study. PLoS Med.

[57] Fisher JA (2018). Healthy volunteers’ perceptions of the benefits of their participation in phase I clinical trials. J Empir Res Hum Res Ethics.

[58] Devine EG (2013). Concealment and fabrication by experienced research subjects. Clin Trials.

[59] Lynch HF (2019). Association between financial incentives and participant deception about study eligibility. JAMA Netw Open.

[60] Lee CP (2018). Deception in clinical trials and its impact on recruitment and adherence of study participants. Contemp Clin Trials.

[61] Ambuehl S, Ockenfels A, Roth AE (2020). Payment in challenge studies from an economics perspective. J Med Ethics.

[62] Krutsinger DC (2020). A randomized controlled trial of behavioral nudges to improve enrollment in critical care trials. Ann Am Thorac Soc.

[63] Jao I (2015). Involving research stakeholders in developing policy on sharing public health research data in Kenya: views on fair process for informed consent, access oversight, and community engagement. J Empir Res Hum Res Ethics.

